# Left Bundle Branch Area Pacing in Transthyretin Cardiac Amyloidosis: A Narrative Review

**DOI:** 10.3390/jcm15010305

**Published:** 2025-12-31

**Authors:** Maria Herrera Bethencourt, Arnt V. Kristen, Vincent Algalarrondo, Guram Imnadze, Andreas Müssigbrodt

**Affiliations:** 1 Department of Cardiology, CHU Martinique (University Hospital of Martinique), 97200 Fort de France, France; maria.herrerabethencourt@chu-martinique.fr; 2 Department of Cardiology, Angiology, Respiratory Medicine, Medical University of Heidelberg, 69120 Heidelberg, Germany; kristen@kardio-darmstadt.de; 3 Cardiovascular Center Darmstadt, 64287 Darmstadt, Germany; 4 Service de Cardiologie, Hôpital Bichat, Unité de Rythmologie Centre de Reference Amylose CERAMIC-CARDIO, 46 rue Henri Huchard, 75018 Paris, France; vincent.algalarrondo@aphp.fr; 5 Department of Rhythmology, University Hospital Ruppin-Brandenburg, Brandenburg Medical School Theodor Fontane, 16816 Neuruppin, Germany; g.imnadze@ukrb.de; 6 Faculty of Medicine, University of Leipzig, 04103 Leipzig, Germany

**Keywords:** cardiac amyloidosis, ATTR, pacemaker, left bundle branch area pacing, LBBAP

## Abstract

**Background/Objectives**: Transthyretin cardiomyopathy (ATTR-CM) is frequently associated with conduction disease requiring pacing. Conventional right ventricular pacing may worsen cardiac function, whereas left bundle branch area pacing (LBBAP) aims to preserve physiological activation. Evidence for LBBAP in ATTR-CM remains limited. **Methods**: A structured narrative review of PubMed and Google Scholar was performed through November 2025 using predefined terms related to LBBAP and ATTR-CM. Peer-reviewed articles, case reports, case series, and relevant abstracts were included. Studies exclusively on light-chain cardiac amyloidosis were excluded. **Results**: Ten publications met inclusion criteria, comprising three case reports, five case series, one retrospective cohort without a comparator, and one cohort comparing LBBAP with cardiac resynchronization therapy (CRT). In total, 56 patients with ATTR-CM underwent LBBAP. Implantation success was high, with stable acute and mid-term electrical parameters. Follow-up (typically 3–12 months) showed stable electrical parameters with narrow paced QRS complexes and preserved or improved left ventricular ejection fraction in most reports. Symptomatic improvement and reductions in natriuretic peptides were variably described. No major lead-related complications were reported. Comparative data remain sparse and inconclusive. **Conclusions**: This review suggests that LBBAP is a feasible and safe pacing approach in patients with ATTR-CM and may help to stabilize or improve heart failure symptoms. Further prospective studies are needed to confirm its clinical effectiveness.

## 1. Introduction

**Figure 1:**
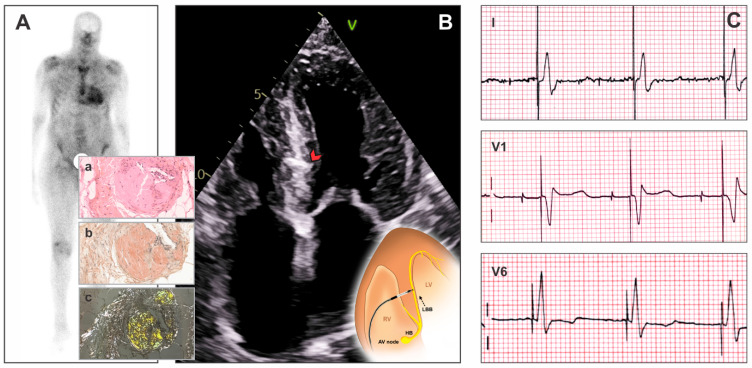
Illustration of diagnosis and treatment in patients with ATTR-CM. (**A**) Bone scintigraphy: intense uptake in the cardiac area, with intensity greater than the costal framework (grade 3 uptake), as typical in ATTR-CM (adapted after [2]). (**a**) Histopathological examination of a fat pad biopsy from the pacemaker pocket in a patient with hereditary transthyretin ATTRv (p.Val142Ile) cardiac amyloidosis. Standard HES (hematoxylin–eosin–saffron) staining of thoracic fat pad biopsy. Fibro-adipose tissue without atypia, showing amyloid deposits in the vascular walls as amorphous eosinophilic extracellular deposits. (**b**) Congo red staining of thoracic fat pad biopsy. Fibro-adipose tissue without atypia, showing amyloid deposits stained with Congo red. (**c**) Amyloid deposits displaying yellow-green birefringence under polarized light (on Congo red-stained slides; adapted after [2]). (**B**) Apical four-chamber view in echocardiography showing the LBBAP lead (red arrow) traversing the interventricular septum. It also shows concentric left ventricular hypertrophy with increased septal echogenicity (“septal brightness”), as typical in CA. Lower corner: schematic illustration of left bundle branch pacing. (**C**) ECG of a patient with ATTR-CM with atrio-ventricular stimulation. Qr pattern in V1 and narrow QRS (115 ms) demonstrating LBBAP. Abbreviations: ATTR-CM, transthyretin cardiomyopathy; LBBAP, left bundle branch area pacing.

Transthyretin (ATTR) amyloidosis is a systemic disorder characterized by the misfolding of transthyretin protein and deposition of insoluble fibrils in body tissues, with a marked predilection for cardiac involvement (Figure 1) [1]. ATTR-cardiomyopathy (ATTR-CM) includes a hereditary variant (ATTRv-CM), caused by pathogenic TTR mutations, and wild-type (ATTRwt-CM), an age-related form without genetic alteration. Both subtypes lead to extracellular deposition of misfolded transthyretin fibrils and progressive cardiac dysfunction. ATTRv-CM is especially prevalent among individuals of Afro-Caribbean descent and is a major cause of progressive heart failure (HF) in older adults, ultimately leading to increased morbidity and mortality [2,3]. Amyloid light-chain cardiomyopathy (AL-CM) arises from the deposition of misfolded immunoglobulin light chains and is associated with a worse prognosis, with median survival often under two years [4].

The accumulation of insoluble amyloid proteins within the myocardium distorts the normal architecture of cardiac myocytes and conduction pathways, leading to marked structural disruption across all forms of cardiac amyloidosis. In addition, mechanisms such as direct cytotoxicity, inflammation, oxidative stress, and apoptosis may contribute to disease development, with each amyloidosis subtype involving a distinct combination of these processes [5]. When ATTR fibrils infiltrate cardiac tissue, they disrupt myocardial mechanics, resulting in impaired systolic contraction and diastolic filling [1]. Cardiac conduction system involvement frequently results in bradyarrhythmias—including sick sinus syndrome and various degrees of atrioventricular (AV) block—as well as tachyarrhythmias like atrial fibrillation [1,5,6]. Disease-modifying therapies such as transthyretin stabilizers (e.g., tafamidis) can slow progression, alleviate symptoms, and improve survival, but no curative treatment for ATTR amyloidosis is currently available [7]. The effect of specific anti-amyloid therapies on cardiac arrhythmias has not been established.

Significant bradyarrhythmias in these patients are generally managed with pacemaker implantation along guideline recommendations [1,5,8]. However, progressive conduction disease and high pacing dependence raise specific management challenges in ATTR-CM. Standard right ventricular pacing (RVP) increases the risk of pacing-induced cardiomyopathy (PIC) and may further worsen heart failure in up to 30% of patients without known ATTR-CM by causing non-physiological ventricular activation [5,9]. This risk may be particularly pronounced in ATTR-CM, as pre-existing diastolic and systolic dysfunction and high RVP burden correlate with an increased risk of pacemaker-induced cardiomyopathy (PIC) [5,6,8,9,10].

Conduction system pacing—especially left bundle branch area pacing (LBBAP)—has gained increased popularity for its ability to preserve near-physiological ventricular activation and minimize the risk of PIC compared to conventional RVP (Figure 1) [9,10]. Preliminary evidence indicates that LBBAP is feasible in patients with ATTR-CM, but prospective comparative studies remain needed to ascertain its long-term benefit over traditional pacing modalities [6,11].

## 2. Methods

**Figure 2:**
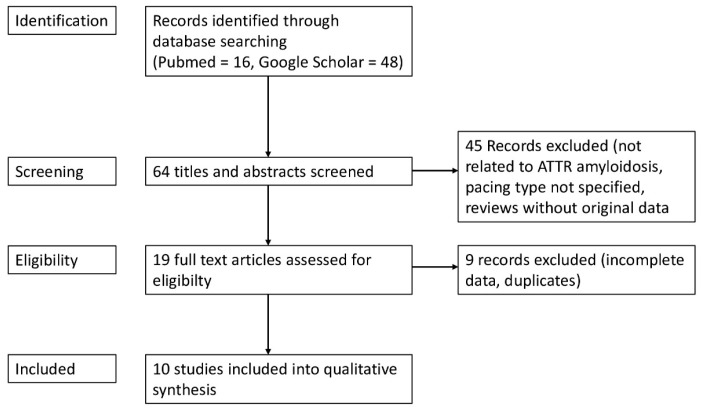
Simplified PRISMA chart illustrating the structured literature search.

A structured literature review was performed to identify publications addressing left bundle branch area pacing (LBBAP) in ATTR-CM. Given the limited evidence—restricted to small case series and individual case reports—a structured narrative rather than a systematic review design was chosen. This approach allowed for a broader interpretation of technical feasibility and clinical relevance based on the currently available data. Searches were conducted in PubMed and Google Scholar, and the selection process is summarized in a simplified PRISMA flowchart (Figure 2). Search terms included combinations of left bundle branch pacing, left bundle branch area pacing, LBBAP, LBBP, transthyretin, ATTR, and cardiac amyloidosis. Reference lists of included papers were screened to identify additional relevant studies.

Peer-reviewed articles, case reports, and case series published in English were eligible. When no full-text publication was available, relevant conference abstracts were included. Studies exclusively on light-chain cardiac amyloidosis were excluded. Study selection was conducted independently by two authors (M.H.B. and A.M.), and any disagreements were resolved by consensus. The search was current as of November 2025.

## 3. Results

A structured literature search was conducted in PubMed and Google Scholar (Figure 1). Studies reporting exclusively on light-chain amyloidosis were excluded. Studies with mixed amyloidosis populations were included when ATTR-CM patients represented the majority. Following the screening process, ten studies met the inclusion criteria and were incorporated into the final analysis (Figure 1 and Table 1). The included publications consisted of three case reports, five case series, one retrospective cohort study without a comparator group, and one retrospective cohort study with a comparator group (Table 1). Two studies were published as abstract only. In total, 56 cases of ATTR-CM treated with LBBAP in ATTR-CM were identified (Table 1).

In 2020 Ahmed et al. published the first case report of LBBAP in a 75-year-old patient with ATTR-CM, with AF and alternating bundle branch block [12]. Following LBBAP, the patient’s clinical status improved from NYHA class III to class II, accompanied by a reduction in NT-proBNP levels at the three-month follow-up (FU) compared with baseline [12]. The paced QRS duration achieved was 105 ms [12].

In 2022 Bermúdez-Jiménez et al. presented a small series of three patients with ATTR-CM and systolic heart failure [13]. Two of these three patients had previously received CRT (cardiac resynchronization therapy) pacemakers but subsequently developed heart failure symptoms resembling pacing-induced cardiomyopathy (PIC) [13]. LBBAP resulted in substantially narrower paced QRS complexes—measuring 128 ms, 138 ms, and 148 ms, respectively—when compared with their intrinsic QRS durations [13]. All patients experienced an improvement in functional status three months after LBBAP implantation, while two patients improved the left ventricular ejection fraction [13].

In 2023 Sudo et al. described a case of LBBAP in a patient with ATTR-CM and mildly reduced LVEF and with stable electrical parameters, improved NYHA functional status, BNP, and LVEF at 3 months FU [14]. The same author described a case of LOT-CRT (left bundle branch area pacing optimized cardiac resynchronization therapy) with improved NYHA functional status, increased LVEF, and decreased NT-proBNP at the FU [15]. LOT-CRT is a pacing strategy that combines LBBAP with coronary sinus branch pacing [21].

Pham-Trung et al. reported 22 successful LBBAP procedures in 23 (78.6 ± 11.7 years), mainly male (82.6%), patients with mildly reduced LVEF (45.5 ± 16.2%) [11]. Twenty patients (87%) had ATTR-CM, whereas three patients (13%) had AL-CM [11]. No procedure-related complications were observed. During the FU of 7.7 ± 5.2 months, one patient with AL-CM died due to disease progression and four patients had to be hospitalized due to decompensated HF [11].

Mirizzi et al. presented a poster at the EHRA Congress in 2024, which has been subsequently published as an abstract [16]. They reported on successful LBBAP in seven patients with improved LVEF at six months FU [16].

Miyajima et al. published a case series of LBBAP in three patients with ATTR-CM with stable electrical parameters, NYHA functional status, and LVEF after 24 and 36 months of FU [17].

Trongtorsak et al. presented an interesting work as a poster at the 2025 HRS Congress, which has been subsequently published as an abstract [18]. They retrospectively compared classic biventricular CRT with LBBAP in patients with CA. Among 54 patients (mean age 79 years), 17 underwent LBBAP and 34 BVP [18]. Baseline characteristics—including conduction disturbances, LVEF, and LV size—were comparable, though LBBAP patients had narrower intrinsic QRS durations [18]. LBBAP implantation succeeded in 80%, producing paced QRS narrowing and physiological LV activation times [18]. During FU, changes in QRS duration, LVEF, and LVEDD did not differ significantly between groups [18]. Rates of the combined endpoint of death or heart failure hospitalization were also similar (35.3% vs. 53.1%) [18].

Sudo et al. described an innovative method of combined endomyocardial biopsy (EMB) and LBBAP [19]. Among 20 cases of EMB with subsequent LBBAP they identified 4 patients with CA [19]. As this work focuses on EMB, only a few details are reported on LBBAP [19].

Finally, Mehta et al. published three cases of successful LBBAP in elderly patients with ATTR-CM [20]. Two patients had a FU of 18 months with stable electrical and favorable echocardiographic parameters [20]. One 91-year-old patient died due to frailty [20].

## 4. Discussion

Since the initial report by Huang and colleagues in 2017, left bundle branch area pacing (LBBAP) has become a recognized first-line conduction system pacing technique in many institutions [22]. LBBAP includes different types of conduction system pacing, e.g., LBBP (left bundle branch pacing), LFP (left fascicular pacing), and LVSP (left ventricular septal pacing) [9]. DSP (deep septal cases) occurs when capture of the conduction system cannot be achieved or is lost during FU [9]. LBBAP overcomes several shortcomings observed with His-bundle pacing, including higher and unstable capture thresholds and the issue of atrial oversensing [9,22]. Only one case report describes the His-bundle pacing in ATTR-CM [23]. In contrast to conventional right ventricular pacing, LBBAP appears to preserve a more physiological activation pattern and may, therefore, reduce the risk of pacing-induced ventricular remodeling [9]. These advantages have prompted growing interest in applying LBBAP to ATTR-CM. To date, the overall encouraging experience with LBBAP in this population is based on ten published reports.

Across these studies, procedural feasibility emerges as a consistent strength. A high implantation success rate of 95.7% (22 of 23 patients) was achieved in the largest single-center series of LBBAP in ATTR-CM [11], while in another cohort 80% of LBBAP attempts were successful [18]. Case reports and small series similarly show that transthyretin amyloid infiltration does not prevent adequate lead fixation with acceptable acute electrical parameters. Notably, these results were obtained in elderly patients with multiple comorbidities and advanced conduction disease, a population that often presents technical challenges for device implantation. Electrical performance during FU was reassuring. Most publications report narrower paced QRS complexes or marked QRS reduction in patients with baseline conduction delay, indicating effective engagement of the conduction system. Capture thresholds and sensing values remained stable when reported, and no major lead-related complications were described. These observations are particularly relevant in ATTR-CM, where progressive infiltration and fibrosis might be expected to compromise long-term lead behavior. Clinical outcomes, although heterogeneously documented, generally suggest stabilization or improvement. Several reports describe a better NYHA functional class after LBBAP, especially in patients with initially impaired LVEF and PIC. By echocardiography, LVEF either improved or remained stable across most reports. Data on natriuretic peptides are limited, but the available observations report reductions or at least stable values over FU. Taken together, these findings indicate that LBBAP may help prevent further hemodynamic decline in a population at risk of PIC (impaired or borderline LVEF, ventricular pacing burden > 40%). In the only comparative cohort, changes in QRS duration, echocardiographic parameters, and the combined endpoint of death or heart failure hospitalization appeared broadly similar between LBBAP and CRT, although the study was small, observational, and included AL-CM [18]. The technique also proved reproducible across various centers. Procedures were performed by different operators, using various lead types and delivery tools, yet the procedural and electrical results were consistent. One series additionally demonstrated that LBBAP can be combined with endomyocardial biopsy during the same procedure, suggesting that both diagnosis and treatment may be streamlined in selected patients [19].

Despite these encouraging findings, several limitations must be acknowledged. The total number of ATTR-CM patients treated with LBBAP remains small, and data derive only from retrospective studies, case series, or single-patient reports, making selection and publication bias likely. Some cohorts [11,16,18] include both ATTR-CM and AL-CM, limiting disease-specific conclusions in a population with a high competing risk of death due to age and disease burden. These studies [11,16,18] reported in total six cases of LBBAP in AL-CM, whereas the majority of LBBAP recipients (*n* = 23, *n* = 7, and *n* = 17, respectively) were diagnosed with ATTR-CM. Outcomes cannot be attributed to the pacing strategy alone, as age, comorbidities, disease-modifying treatments such as transthyretin stabilizers (e.g., tafamidis), and heart failure therapies all influence disease progression and competing risks of morbidity and mortality. This limitation further highlights the need for prospective, controlled studies. FU duration is generally short, often between three and twelve months, and may not capture long-term changes in ventricular function, disease progression, or long-term lead performances. Reporting of pacing-related details is also incomplete, regarding the programmed pacing mode, lower pacing rate, or the percentage of ventricular pacing, which affect interpretation. Another limitation is the inconsistent reporting of the paced QRS morphology, to distinguish between LBBP, LFP, LVSP, and DSP.

Optimal patient selection is essential when considering pacing strategies in ATTR-CM. LBBAP may be particularly suitable for patients with a high anticipated pacing burden due to advanced conduction disease or second- and third-degree atrioventricular block [9]. It also represents a reasonable option for patients with narrow or mildly prolonged QRS complexes who do not meet conventional criteria for CRT but remain at risk of pacing-induced deterioration [9]. Furthermore, patients with borderline or reduced left ventricular ejection fraction (LVEF) may benefit from the preservation of electromechanical synchrony afforded by LBBAP [9]. LBBAP can also serve as a therapeutic upgrade in patients with suspected or confirmed PIC [9]. CRT remains the established treatment for patients with LBBB and reduced LVEF, effectively restoring mechanical synchrony (Table 2) [5,6,8]. However, its benefit may be limited in patients with narrow QRS duration, non-LBBB conduction abnormalities, or suboptimal coronary venous anatomy [8,9]. LBBAP may be considered as an alternative in cases where coronary sinus branches are unsuitable for CRT [9]. LOT-CRT is a pacing strategy that combines LBBAP with coronary sinus branch pacing and has shown potential benefit over stand-alone CRT or stand-alone LBBAP in selected patients, particularly those with a more advanced NYHA functional class, larger left ventricles, and greater scar burden (Table 2) [21]. RVP remains technically simple, widely available, and associated with low and stable pacing thresholds (Table 2) [6]. However, its non-physiological activation pattern can induce or worsen ventricular desynchrony and promote PIC—effects that may be particularly deleterious in the restrictive, infiltrated myocardium of ATTR-CM (Table 2) [6,12]. His-bundle pacing (HBP) offers the most physiological ventricular activation by directly recruiting the His–Purkinje system, but its use in infiltrative cardiomyopathies is often limited by high or unstable thresholds, risk of oversensing or loss of capture, and technical challenges related to septal fibrosis (Table 2) [23]. LBBAP represents an attractive compromise, achieving distal engagement of the left bundle branch with generally lower and more stable thresholds. This approach results in narrower paced QRS complexes, more physiological ventricular activation, and potentially reduced risk of PIC, helping to preserve ventricular function (Table 2) [9]. As an important limitation, much of the presumed benefit of LBBAP is extrapolated from the literature in non-amyloid populations (Table 2) [9,10].

Overall, the available literature suggests that LBBAP in ATTR-CM is feasible and safe. It also seems to be efficient, as it is associated with clinical stabilization or improvement in patients with ATTR-CM who require pacing. However, given the small and heterogeneous nature of the evidence, these conclusions should be interpreted with great caution. Larger multicenter registries and prospective, adequately powered studies are needed to clarify optimal patient selection, long-term safety, and the comparative role of LBBAP relative to conventional right ventricular pacing and CRT. Until such data are available, the choice of pacing modality in ATTR-CM should be individualized, taking into account disease stage, anatomical considerations, coexistence of heart failure therapies, frailty, and operator expertise.

## 5. Conclusions

This review suggests that LBBAP is a feasible and safe pacing approach in patients with ATTR-CM and may help to stabilize or improve heart failure symptoms. Further prospective studies are needed to confirm its clinical effectiveness.

## Figures and Tables

**Table 1:** Main findings of case reports and studies on LBBAP in ATTR-CM. Abbreviations: AF, atrial fibrillation; AL-CM, amyloid light-chain cardiomyopathy; ATTR-CM, transthyretin cardiomyopathy; BVP, biventricular pacing; BNP/NT-proBNP, (N-terminal pro) B-type natriuretic peptide; CA, cardiac amyloidosis; CS, coronary sinus; CRT, cardiac resynchronization therapy; DDD/DDDR, dual-chamber pacing (with/without rate response); LVEF, left ventricular ejection fraction; FU, follow-up; GLS, global longitudinal strain; HF, heart failure; LOT-CRT, left bundle branch area pacing–optimized CRT; LBBAP/LBBP, left bundle branch area pacing/left bundle branch pacing; LPR, left pacing rate; LV, left ventricle; LVEDD, left ventricular end-diastolic diameter; NYHA, New York Heart Association class; PIC, pacing-induced cardiomyopathy; RVP, right ventricular pacing; wtATTR/vATTR, wild-type/variant ATTR; non-specified ATTR, ATTR without genetic testing results; VVIR, ventricular pacing with rate response.

Author (Year)	Study Type	Sample Size	AF (%)	LVEF at Implantation	LBBAP Lead	Pacing Mode and LPR	Ventricular Pacing (%)	Complications	Follow-Up (Months)	Main Finding
Ahmed et al. (2020) [12]	Case Report	*n* = 1: wtATTR-CM	100%	55%	SelectSecure 3830	Not reported	Not reported	None	6	Stable electrical parameters. Improved NYHA, NT-proBNP, and GLS
Bermúdez-Jiménez et al. (2022) [13]	Case series	*n* = 3: wtATTR-CM *n* = 2, vATTR *n* = 1	100%	37% 43% 30%	SelectSecure 3830	VVIR DDD DDDR LPR not reported	97% 99.6% 99.9%	None	3	Stable electrical parameters. Improved NYHA and BNP in 3 cases, improved LVEF in 2 cases, stable LVEF in 1 case
Sudo et al. (2023) [14]	Case Report	CA *n* = 1: ATTR-CM	100%	46%	SelectSecure 3830	DDD(R) LPR not reported	Not reported	None	3	Stable electrical parameters. Improved NYHA, BNP, and LVEF
Pham-Trung et al. (2023) [11]	Retrospective cohort study	*n* = 23 (successful LBBAP in 22 patients) ATTR *n* = 20 AL *n* = 3	74%	45.5 ± 16.2%	SelectSecure 3830 *n* = 6 Solia S *n* = 17	VVIR DDD LPR not reported	96%	None	7.7 ± 5.2	Stable electrical parameters, NT-pro BNP, and LVEF. 1 death (AL-CM), 4 patients with decompensated HF
Sudo et al. (2024) [15]	Case Report	*n* = 1: ATTR-CM	100%	42%	SelectSecure 3830 with CS branch pacing (LOT-CRT)	Not reported	Not reported	None	Not reported	Stable electrical parameters. Improved NYHA, NT-proBNP, and LVEF
Mirizzi et al. (2024) [16]	Case series	*n* = 7: wtATTR *n* = 5, AL *n* = 2	43%	45 ± 6%	Stylet driven leads	Not reported	Not reported	None	6	Improved LVEF
Miyajima et al. (2024) [17]	Case series	*n* = 3 wtATTR *n* = 2, vATTR *n* = 1	33%	64% 59% 52%	SelectSecure 3830 Solia S Ingevity	Not reported	Not reported	None	24–36	Stable electrical parameters, stable NYHA status, stable LVEF
Trongtorsak et al. (2025) [18]	Retrospective cohort study	*n* = 54: LBBAP: 17 (6 vATTR, 10 wtATTR, 1 AL) BVP: 34 (10 vATTR, 19 wtATTR, 8 AL).	Not reported	46.9 ± 10% in LBBAP 44.1 ± 10.5% in CRT	Not reported	Not reported	Not reported	Not reported	31.3 ± 25.6	Stable LVEDD and LVEF in LBBAP and CRT patients. Composite endpoint of death or HF: 35.3% in LBBAP and 53.1% in CRT (*p* = 0.99)
Sudo et al. (2025) [19]	Case series	*n* = 20, ATTR *n* = 3, non-specified *n* = 1	Not reported	Not reported	Not reported	Not reported	Not reported	None	3.4 ± 2.4	Endomyocardial biopsy is feasible with 3D delivery sheaths during device implantation procedures
Mehta et al. (2025) [20]	Case series	*n* = 3: non-specified ATTR	33%	30–35% 55–60% 60–65%	SelectSecure 3830	Not reported	Not reported	None	18 in 2 cases died after 3 months	Stable electrical parameters and LVEF in 2 cases. Stable electrical parameters at 6 weeks follow-up in 1 case
In Total	*n* = 10 3 case reports, 5 case series, 2 retrospective cohort studies	*n* = 56 LBBAP in ATTR-CM *n* = 6 LBBAP in AL-CM	33–100%	30–65%	SelectSecure 3830 Solia S Ingevity	VVI(R) DDDR LPR not reported	96–99.9% Not reported in majority of studies	None reported	3–36 months	Stable electrical parameters, stable or improved NYHA status, stable or improved LVEF

**Table 2:** Comparison of general aspects between different pacing strategies, extrapolated from the literature in non-amyloid populations and its validation in ATTR-CM. RVP: right ventricular pacing; HBP: His-bundle pacing; LBBAP: left bundle branch area pacing; CRT: cardiac resynchronization therapy; LOT-CRT: left bundle branch area pacing optimized cardiac resynchronization therapy; ATTR-CM: transthyretin cardiomyopathy. (+1) *: +1 ventricular lead if additional defibrillator lead or right ventricular pacing lead is required.

	Number of Ventricular Leads	Technical Difficulty	Ventricular Synchrony	Long-Term Performance	Validation in ATTR-CM
RVP	1	+	-	+++	Historical reference
HBP	1 (+1) *	+++	+++	+	No data
LBBAP	1 (+1) *	++	++	++	Few series
CRT	2	++	++	++	Few series
LOT-CRT	2 (+1) *	++&++	+++?	++	One case

## Data Availability

The original contributions presented in this study are included in the article. Further inquiries can be directed to the corresponding author.
